# Risk factors of substance use treatment gaps among a nationally representative sample of black American adults in relation to sexual minority status and health insurance coverage

**DOI:** 10.1186/s40359-023-01352-7

**Published:** 2024-05-15

**Authors:** Josiah K. Rice, Kaston D. Anderson-Carpenter, Javon D. Ellis

**Affiliations:** Department of Psychology, Michigan State University, 316 Physics Road, East Lansing, MI 48824 USA

**Keywords:** Black american, Treatment gap, Sexual minority, Substance use, Health insurance

## Abstract

**Background:**

Little research has investigated predictors of specialty substance use treatment gaps among Black adults. This study examined differential odds of experiencing self-reported, past-year treatment gaps among Black adults with respect to sexual minority status and health insurance coverage, accounting for social cofactors.

**Method:**

This cross-sectional study comprised 36,098 Black Americans aged 18 and older who completed the 2015–2019 National Survey on Drug Use and Health (NSDUH) and provided responses for all selected survey items. Design-based multivariable logistic regression models were used to examine predictors of drug and alcohol treatment gaps.

**Results:**

Sexual minority Black adults reported greater odds of experiencing treatment gaps to specialty treatment (i.e., inpatient hospital, inpatient/outpatient rehabilitation facility, or mental health center) compared to Black heterosexuals in adjusted models (Gay or lesbian: AOR = 2.01, 95% CI = 1.39–2.89; Bisexual: AOR = 2.35, 95% CI = 1.77–3.12), with bisexual Black women experiencing the greatest odds (AOR = 3.10, 95% CI = 2.33–4.14). Black adults with no health insurance were significantly more likely to report substance use treatment gaps relative to their peers with health insurance coverage (AOR = 50, 95% CI = 1.26–1.78).

**Conclusion:**

The results suggest a critical need for more investigations into patterns of specialty substance use treatment gaps within Black populations and for developing sexual identity-affirming mechanisms for closing the disparity gap, particularly for Black sexual minorities and those who lack health insurance coverage.

## Introduction

Substance use treatment gaps (i.e., needing substance use treatment services but not receiving them) exacerbate the deleterious effect of substance misuse on adults, particularly among Black Americans. Black adults enter substance use treatment at an older age, with lower socioeconomic statuses, and with a wider range of self-reported primary substance use relative to their White counterparts [1]. Not only do Black patients comprise the highest proportion of racial/ethnic groups to participate in alcohol use disorder treatment compared to other racial/ethnic groups, but they also experience significantly greater odds for initiating AUD treatment compared to their White counterparts [2]. Some research suggests that Black adults with substance use disorder still report lower odds of using any substance use treatment services compared to their White peers, even after controlling for sociodemographic characteristics, problem severity, and perceived treatment need [3–7]. Furthermore, Black Americans’ participation in treatment services more often include 12-step programs and spiritual providers than specialized treatment [8].

Gender disparities also exist in substance use treatment utilization, [9, 10] further widening the treatment gap among Black Americans. Studies have shown that women are more likely than men to report that substance use conditions will resolve by themselves [11, 12]. However, Black women are not only more likely to receive inpatient treatment than Black men, but they are also more likely to have a lower engagement in counseling [13]. Nevertheless, Black women still experience particularly noticeable rates of unmet substance use treatment needs, with approximately 43% experiencing a substance use treatment gap [^14 15^]. Thus, reducing the race- and gender-related disparities that Black women uniquely experience in accessing and utilizing substance use treatment requires the need for centering Black womanhood and familial empowerment in providing services.

### Sexual minority status and substance use treatment

Extensive research [16–18] suggests that sexual minority (i.e., lesbian, gay, and bisexual) adults experience greater substance use rates than heterosexuals. Although sexual minority adults report greater substance use compared to their heterosexual peers, bisexual adults specifically have significantly greater odds of lifetime substance use treatment utilization [17]. Data on substance use treatment utilization, however, paint a more nuanced and inconsistent picture for sexual minorities. Some findings suggest that bisexual adults experience significantly lesser odds of utilizing substance use treatment services than their heterosexual counterparts; [19] others, however, indicate that bisexual women report greater odds of treatment utilization compared to heterosexual women [20]. Despite these gender effects, bisexual adults overall experience even greater barriers to substance use treatment compared to other sexual minorities [21]. Thus, nuances in substance use treatment engagement within and across sexual minority subgroups suggest that minority stressors may affect these communities in unique ways that warrant further investigation.

For Black sexual minority adults, the intersection of race and sexual minority status exacerbate substance use and treatment engagement. Black bisexual women, regardless of age, are more than twice as likely to report past-year illicit drug use, alcohol use disorder, and/or substance use disorder compared to Black heterosexual women [22]. Stigma remains a key influencing factor of elevated substance use rates among sexual minorities, with sexual minority-related stigma and discrimination being associated with greater risk of non-alcohol substance use, especially for bisexual individuals even after controlling for race/ethnicity [23, 24]. Thus, understanding differential risks for experiencing a substance use treatment gap may necessitate more nuanced examinations accounting for sexual orientation.

### Health insurance and substance use treatment

Having public or private health insurance may support engagement in specialty substance use treatment (i.e., formal programs such as inpatient/outpatient and rehabilitation services) [25, 26]. However, disparities in health insurance coverage exist despite local, state, and national efforts to increase coverage. For example, Black adults endorsing lower rates of health insurance coverage compared to their White peers, [27, 28] and sexual minority adults continue to experience challenges in receiving substance use treatment services covered by health insurance [29]. Although data for Black sexual minority adults remains sparse, they may experience even greater challenges in accessing insurance-covered substance use treatment services than Black heterosexual or White sexual minority adults alone.

It is worth noting, however, that the influence of health insurance on specialty substance use treatment services may depend on the type of insurance. Some individuals with Medicaid were more likely to receive substance use treatment compared to those with private insurance;^26^]. and results from other samples indicate that having Medicare or Medicaid—and not private insurance—was significantly associated with specialty substance use treatment utilization [25]. Socioeconomic factors, such as education and household income, may also affect the type of insurance Black Americans use. Thus, insurance type, along with the socioeconomic differences related to type of insurance, may play a role in utilization of substance use treatment services among Black Americans, particularly those with multiple intersecting identities.

### Theoretical framework and hypotheses

This study is grounded in the minority stress model [30–32] and intersectionality theory [33, 34]. According to these perspectives, sexual minority individuals experience unique stressors that negatively influence psychosocial and health outcomes. Intersectionality posits that stressors are often compounded due to intersecting identities in complex, nuanced, and often counterintuitive mechanisms. For example, Demant et al. [35] found that racial/ethnic minority women who identified as sexual minorities showed no elevated risk for high-risk alcohol consumption compared to their White heterosexual peers. However, their findings indicated differential risks for substance use across various other illicit substances. Intersectionality particularly centers on understanding the role of oppressive systems (e.g., sexism, misogynoir, racism, heterosexism, capitalism) in the lives and well-being of Black Americans, whereas the minority stress model highlights the role of personal intersecting identities in health outcomes. Together, the two theoretical positions suggest that subgroups of Black Americans experience unique and compounded barriers to substance use treatment that are influenced by oppressive systems such as racism, heterosexism, and misogynoir.

To date, few studies have investigated influencing factors of substance use treatment gaps within Black American populations specifically. Given previous findings, [6, 27, 36] we hypothesized that Black bisexual adults would experience greater odds of both past-year alcohol and past-year drug use treatment gap relative to Black lesbian, gay, and heterosexual adults after adjusting for social cofactors. We also hypothesized that compared to Black adults with health insurance, those with no health insurance would experience greater odds of a past-year specialty alcohol and drug use treatment gaps after adjusting for social cofactors.

## Material and method

### Participants

Participants for this cross-sectional, secondary data analysis comprised 36,098 noninstitutionalized Black Americans aged 18 and older who completed the 2015–2019 National Survey on Drug Use and Health (NSDUH) and provided responses for all selected survey items [37–42]. The NSDUH employs a state-based design with an independent, multistage area probability sample within each state and the District of Columbia. In addition, sensitive questions in the NSDUH are completed using audio computer-assisted survey interviewing which has shown to increase response rates of certain sensitive questions [43]. The weighted interview response rates for the 2015–2019 surveys were 69.25%, 68.14%, 67.12%, 66.56%, and 64.92% respectively. This study was exempt from ethical review by the Michigan State University IRB, and all methods in this secondary analysis were carried out in accordance with relevant guidelines and regulations.

### Variables and measures

Outcome variables were past-year (i.e., past 12 months) specialty alcohol and drug treatment gap. Specialty treatment gap was a self-reported variable and defined as needing treatment for alcohol or illicit drug use in the past year but not receiving it at a specialty facility (i.e., inpatient hospital, inpatient or outpatient rehabilitation center, or mental health center). Drug use was defined as using any of the following substances: marijuana, cocaine, heroin, hallucinogens, inhalants, methamphetamine, prescription pain relievers, tranquilizers, stimulants, or sedatives. For the purposes of this study, we included marijuana as an illicit drug for two reasons: (1) it is included as part of “illicit drug use” classification in the NSDUH, and (2) not all states have legalized or decriminalized recreational marijuana use at the time of this writing.

Both outcome variables were measured separately and dichotomously (i.e., yes/no). Predictor variables included age (18–34 years, 35–49 years, 50 years or older); sexual orientation (heterosexual, gay or lesbian, bisexual); education (college graduate, associate’s degree, some college, high school/GED, less than high school); poverty level (more than twice the federal poverty line, up to twice the federal poverty line, living in poverty); marital status (married, single, separated or divorced, widowed); and health insurance (yes or no). Gender (men or women) was included as a sub-group variable.

### Data analysis

Given the nature of the complex survey design, we used Stata’s svyset command to apply appropriate weights to account for multiple years. After pooling data from the 2015–2019 NSDUH, we generated a new weight variable to equal the pooled weight divided by five to account for the survey data being pooled across years. The new weight variable was then used in setting survey weights in the *svyset* command. Missing data analysis indicated that sexual orientation (25.6%), education (24.1%), and marital status (29.7%) were missing at random in the dataset. Therefore, we conducted analyses using multiple imputation by chained equations (MICE) with 15 imputations. MICE was decided as an appropriate statistical method for this study given its flexibility with varying types of data such as continuous and dichotomous variables [44]. To identify significant predictors of experiencing a specialty alcohol or drug treatment gap among Black adults, we first used design-based chi-square tests to examine significant bivariate associations in the total sample and stratified by gender. Predictor variables with α < 0.05 were then entered into separate design-based multivariable logistic regression models for past-year alcohol and past-year drug use treatment gap, respectively. Results were reported as crude and adjusted exponentiated log-odds and 95% confidence intervals, with statistical significance for multivariable models also set at α < 0.05. All analyses were conducted using Stata SE Version 15.1 [45].

## Results

### Sociodemographic characteristics

Of the 36,098 Black American adult respondents in the study’s sample, 45.8% comprised men, and 94.9% were heterosexual Black Americans (Table 1). More than half (59.9%) were aged 35 and older, and 34.2% were 50 years old or older. Approximately 83.4% of the overall sample had a high school education or more, and 26.3% were living in poverty. Approximately 67.3% were single, separated or divorced, or widowed; 12.2% of the sample did not have health insurance. There were significant differences between men and women by age, sexual orientation, education, poverty level, marital status, and health insurance (all ps ≤ 0.001).

**Table 1:** Weighted percentages of sociodemographic characteristics by gender (Weighted N = 36,098)

Sociodemographic characteristic	Men (%)	Women (%)	Total (%)	Sig.
Age				
50 years or older	16.3	22.7	40.2	0.001
35–49 years old	11.6	14.1	25.7	
18–34 years old	17.4	17.8	34.2	
Sexual orientation				< 0.001
Bisexual	0.8	2.6	3.4	
Gay or lesbian	1.0	1.1	2.5	
Heterosexual	43.6	50.9	94.5	
Education				< 0.001
Less than high school	8.3	8.4	16.6	
High school/GED	15.7	15.8	31.5	
Some college	10.0	13.7	23.7	
Associate’s degree	3.5	5.1	8.7	
College graduate or higher	7.9	11.7	19.5	
Poverty level				< 0.001
Living in poverty	10.2	16.1	26.3	
Up to 2x poverty line	11.5	14.7	26.2	
> 2x poverty line	23.6	23.9	47.4	
Marital status				< 0.001
Single	20.8	22.4	45.1	
Married	17.0	15.8	32.7	
Separated or divorced	6.2	10.1	16.3	
Widowed	1.4	4.6	5.9	
Health insurance				< 0.001
No	7.0	5.2	12.2	
Yes	38.2	49.6	87.8	

Note. Percentages may not add to 100% due to rounding.

**Fig. 1:**
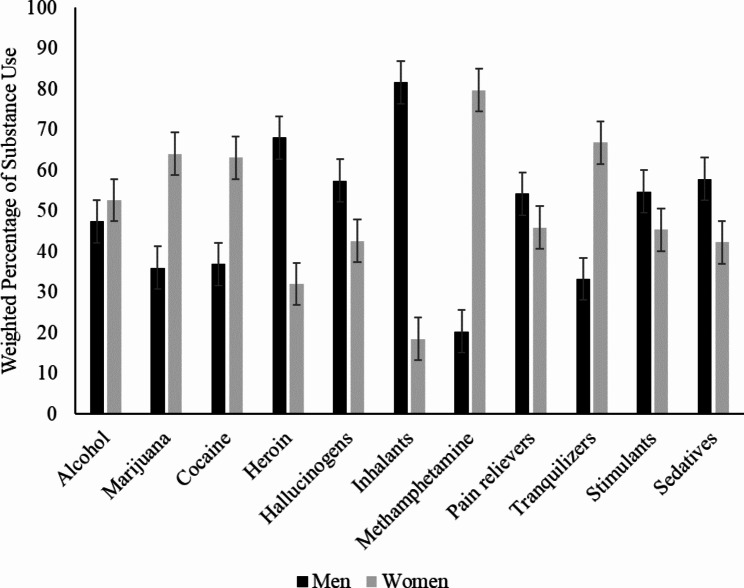
Weighted percentages and type of substance used by Black American men and women (N = 36,098) with 95% CI error bars. Gray bars denote weighted percentages for Black women, and black bars correspond to weighed percentages for Black men

Figure 1 shows the weighted proportional distribution of substances by type as reported by the respondents. Although the pooled proportional means by gender indicate no significant differences (M_men_ = 49.8%, SD_men_ = 17.4%; M_women_ = 50.2%, SD_women_ = 17.4%; p > .05), differences were found in gender comparisons. A significantly greater proportion of Black women reported marijuana, cocaine, methamphetamine, and tranquilizer use compared to Black men (all ps < 0.05). Conversely, a significantly greater proportion of Black men reported heroin, hallucinogen, inhalant, and sedative use compared to Black women (all ps < 0.05).

### Past-year specialty alcohol treatment gap

Table 2 shows the crude and adjusted odds ratios and 95% confidence intervals for experiencing specialty alcohol treatment gap in the past year among Black Americans. Compared to heterosexual adults, Black gay men and lesbian women (AOR: 2.01, 95% CI: 1.39–2.89), as well as Black bisexual adults (AOR: 2.35, 95% CI: 1.77–3.12) experienced greater gaps in specialty alcohol treatment services compared to heterosexuals. Stratified analyses demonstrated elevated odds in both sexual minoritymen (gay—AOR: 2.11, 95% CI: 1.26–3.52; bisexual—AOR: 2.12, 95% CI: 1.14–3.96) and sexual minority women (lesbian—AOR: 1.94, 95% CI: 1.23–3.04; bisexual—AOR: 3.10, 95% CI: 2.33–4.14) relative to their heterosexual counterparts. In the overall sample, adults aged 50 and older reported significantly lower odds of experiencing an alcohol treatment gap (AOR: 0.67, 95% CI: 0.54–0.84). Similarly older Black women reported lower odds of experiencing a specialty alcohol treatment gap compared to their 18-34-year-old counterparts (AOR: 0.47, 95% CI: 0.32–0.68).

**Table 2:** Weighted crude and adjusted odds with 95% confidence intervals of experiencing a past-year gap in specialty alcohol treatment services among Black adults by gender

Sociodemographic characteristic	Men		Women			Total		
	OR (95% CI)	AOR (95% CI)		OR (95% CI)	AOR (95% CI)		OR (95% CI)	AOR (95% CI)
Age								
18–34 years old								
35–49 years old	0.97 (0.82, 1.16)	1.25* (1.03, 1.51)		0.58*** (0.46, 0.75)	0.78 (0.59, 1.03)		0.78** (0.67, 0.91)	1.01 (0.85, 1.20)
50 years or older	0.64*** (0.51, 0.81)	0.88 (0.68, 1.14)		0.29*** (0.21, 0.41)	0.47*** (0.32, 0.68)		0.46*** (0.38, 0.57)	0.67** (0.54, 0.84)
Sexual orientation								
Heterosexual								
Gay or lesbian	2.36** (1.46, 3.82)	2.11** (1.26, 3.52)		2.60*** (1.66, 4.09)	1.94** (1.23, 3.04)		2.46*** (1.73, 3.49)	2.01*** (1.39, 2.89)
Bisexual	1.68 (0.93, 3.01)	2.12* (1.14, 3.96)		4.32*** (3.27, 5.70)	3.10*** (2.33, 4.14)		2.66*** (2.05, 3.45)	2.35*** (1.77, 3.12)
Education								
College graduate								
Some college/Associate’s degree	1.21 (0.92, 1.61)	1.11 (0.81, 1.53)		1.00 (0.72, 1.38)	0.78 (0.54, 1.12)		1.11 (0.90, 1.38)	0.99 (0.78, 1.27)
High school/GED	1.38** (1.09, 1.76)	1.21 (0.90, 1.63)		0.98 (0.73, 1.33)	0.75 (0.55, 1.03)		1.25* (1.02, 1.53)	1.12 (0.89, 1.42)
Less than high school	2.02*** (1.47, 2.78)	1.77** (1.18, 2.66)		0.95 (0.58, 1.55)	0.78 (0.45, 1.34)		1.60*** (1.30, 1.96)	1.52** (1.16, 1.99)
Poverty level								
More than 2x poverty line								
Up to 2x poverty line	0.89 (0.69, 1.13)	0.93 (0.75, 1.16)		0.90 (0.69, 1.19)	1.11 (0.83, 1.49)		0.92 (0.77, 1.11)	0.93 (0.77, 1.12)
Living in poverty	0.80 (0.64, 1.00)	1.01 (0.77, 1.32)		0.79 (0.62, 1.00)	1.09 (0.87, 1.38)		0.85* (0.73, 0.99)	0.90 (0.75, 1.08)
Marital status								
Married								
Single	1.88*** (1.46, 2.43)	1.78*** (1.34, 2.37)		2.82*** (1.99, 3.99)	2.21***(1.52, 3.22)		2.10*** (1.71, 2.58)	1.83*** (1.45, 2.30)
Separated or divorced	1.42 (0.91, 2.20)	1.33 (0.85, 2.08)		1.44 (0.90, 2.32)	1.59 (0.97, 2.62)		1.29 (0.96, 1.74)	1.29 (0.95, 1.74)
Widowed	1.07 (0.50, 2.27)	1.07 (0.52, 2.22)		0.91 (0.44, 1.89)	1.26 (0.58, 2.73)		0.77 (0.47, 1.26)	0.86 (0.52, 1.42)
Health insurance								
Yes								
No	1.99*** (1.55, 2.54)	1.54** (1.20, 1.97)		0.67** (0.53, 0.86)	1.12 (0.88, 1.44)		1.93*** (1.63, 2.29)	1.50*** (1.26, 1.78)

*Note.* *p < .05; **p < .01; ***p < .001

Compared to Black college graduates, Black adults with less than a high school education showed significantly greater odds of experiencing a gap in specialty alcohol treatment (AOR: 1.52, 95% CI: 1.16–1.99). Similar results were not found among Black women, but Black men with less than a high school education reported 1.77 times greater odds (95% CI: 1.18–2.66) of experiencing an alcohol treatment gap compared to Black men who graduate college. Crude analyses in the overall and stratified samples showed that living in poverty was independently associated with lesser odds of experiencing a treatment gap compared to those living at more than twice the federal poverty line (AOR: 0.85, 95% CI: 0.73–0.99; however, the odds were nonsignificant in adjusted models.

In the adjusted models, single Black adults reported significantly greater odds of experiencing a gap in receiving specialty alcohol treatment services compared to their married peers (AOR: 1.83, 95% CI: 1.45–2.30). We found that a single marital status remained a significant predictor for both Black men (AOR: 1.78, 95% CI: 1.34–2.37) and Black women (AOR: 2.21, 95% CI: 1.52–3.32) compared to their married counterparts. Having no health insurance was significantly associated with elevated odds of experiencing a gap in specialty alcohol treatment in the overall sample (AOR: 1. 50, 95% CI: 1.26–1.78) compared to having health insurance of any kind. Similar results were found for Black men (AOR: 1.54, 95% CI: 1.20–1.97) but not for Black women.

### Past-year specialty drug treatment gap

Table 3 shows the crude and adjusted odds ratios and 95% confidence intervals for experiencing a gap in specialty drug treatment in the past year. Black sexual minorities were at an elevated risk of not getting needed specialty drug treatment than their heterosexual peers. Specifically, Black gay and lesbian adults reported almost two-fold greater odds (AOR: 2.45, 95% CI: 1.64–3.66) and Black bisexual adults with more than two-fold greater odds (AOR: 2.17, 95% CI: 1.54–3.06) compared to Black heterosexual adults. In stratified analyses, Black gay men (AOR: 2.71, 95% CI: 1.60–4.60); Black lesbian women (AOR: 2.45, 95% CI: 1.50-4.00); and Black bisexual women (AOR: 3.93, 95% CI: 2.68–5.74) reported greater odds of a past-year specialty drug treatment gap compared to Black heterosexuals.

**Table 3:** Weighted crude and adjusted odds with 95% confidence intervals of experiencing a past-year gap in specialty drug treatment services among Black adults by gender

Sociodemographic characteristic		Men			Women				Total			
		OR (95% CI)	AOR (95% CI)			OR (95% CI)	AOR (95% CI)			OR (95% CI)	AOR (95% CI)	
Age												
18–34 years old												
35–49 years old		0.38*** (0.30, 0.48)	0.51*** (0.39, 0.66)			0.29*** (0.21, 0.41)	0.45*** (0.31, 0.64)			0.34*** (0.28, 0.42)	0.47*** (0.37, 0.59)	
50 years or older		0.30*** (0.20, 0.46)	0.42*** (0.26, 0.67)			0.31*** (0.19, 0.51)	**0.63 (0.34, 1.15)**			0.30*** (0.20, 0.44)	0.46** (0.29, 0.72)	
Sexual orientation												
Heterosexual												
Gay or lesbian		3.02*** (1.86, 4.92)	2.71*** (1.60, 4.60)			3.17*** (1.98, 5.08)	2.45** (1.50, 4.00)			3.08*** (2.17, 4.39)	2.45*** (1.64, 3.66)	
Bisexual		1.29 (0.71, 2.34)	1.27 (0.64, 2.54)			5.31*** (3.77, 7.49)	3.93*** (2.68, 5.74)			2.82*** (2.09, 3.81)	2.17*** (1.54, 3.06)	
Education												
College graduate												
Some college/Associate’s degree		1.52 (1.00, 2.30)	1.25 (0.75, 2.07)			1.68* (1.12, 2.53)	1.31 (0.78, 2.20)			1.60** (1.14, 2.23)	1.31 (0.87, 1.99)	
High school/GED		1.65* (1.06, 2.62)	1.33 (0.75, 2.34)			1.91* (1.17, 3.13)	1.43 (0.75, 2.72)			1.85** (1.28, 2.67)	1.51 (0.94, 2.45)	
Less than high school		2.96*** (1.81, 4.83)	2.47** (1.35, 4.54)			1.89* (1.05, 3.39)	1.51 (0.75, 3.08)			2.71*** (1.81, 4.08)	2.48** (1.47, 4.19)	
Poverty level												
More than 2x poverty line												
Up to 2x poverty line		0.76* (0.59, 0.99)	1.07 (3, 1.39)			0.87 (0.65, 1.17)	1.28 (0.88, 1.86)			0.84* (0.70, 1.00)	1.06 (0.85, 1.33)	
Living in poverty		0.53*** (1, 0.69)	1.30 (0.95 1.78)			0.58** (0.42, 0.80)	1.21 (0.79, 1.85)			0.60*** (0.50, 0.71)	1.07 (0.86, 1.33)	
Marital status												
Married												
Single		3.56*** (2.64, 4.79)	2.29*** (1.64, 3.18)			2.82*** (1.81, 4.37)	1.77* (1.05, 3.01)			3.17*** (2.49, 4.05)	2.02*** (1.54, 2.65)	
Separated or divorced		1.56 (0.88, 2.76)	1.54 (0.84, 2.84)			0.99 (0.51, 1.93)	1.01 (0.50, 2.04)			1.22 (0.81, 1.83)	1.25 (0.80, 1.95)	
Widowed		2.25 (0.71, 7.15)	2.38 (0.68, 8.33)			0.28 (0.05, 1.47)	0.27 (0.05, 1.56)			0.86 (0.32, 2.32)	0.89 (0.31, 2.59)	
Health insurance												
Yes												
No		2.08*** (1.60, 2.70)	1.38 (0.98, 1.93)			1.60* (1.11, 2.31)	1.23 (0.81, 1.89)			2.06*** (1.68, 2.52)	1.49** (1.14, 1.94)	

*Note.* *p < .05; **p < .01; ***p < .001

Overall, adults aged 35 and older showed significantly lower odds of experiencing a drug treatment gap compared to their 18-34-year-old counterparts, with Black Americans aged 50 and older experiencing the lowest odds (AOR: 0.46, 95% CI: 0.29–0.72). Subgroup analyses showed similar findings among Black men, with those aged 50 and older reporting the lowest odds of experiencing a specialty drug treatment gap relative to 18-34-year-olds (AOR: 0.42, 95% CI: 0.26–0.67). Among Black women, those aged 35–49 years old reported the lowest odds of experiencing a drug treatment gap (AOR: 0.45, 95% CI: 0.31–0.64).

Compared to Black college graduates, Black adults with less than a high school education experienced significantly greater odds of experiencing a gap in specialty drug treatment (AOR: 2.48, 95% CI: 1.47–4.19). Comparable results were not found among Black women, but Black men with less than a high school education reported 2.47 times greater odds (95% CI: 1.35–4.54) of experiencing an alcohol treatment gap compared to Black men who graduated college. Adjusted analyses showed that while poverty level was not a significant predictor in the overall or stratified samples, it was a significant predictor in crude analyses.

In the adjusted models, single Black adults reported two-fold greater odds of experiencing a gap in receiving specialty drug treatment services compared to their married peers (AOR: 2.02, 95% CI: 1.54–2.65). Single marital status remained a significant predictor for both Black men (AOR: 2.29, 95% CI: 1.64–3.18) and Black women (AOR: 1.77, 95% CI: 1.05–3.01) compared to their married counterparts. Having no health insurance was significantly associated with elevated odds of experiencing a gap in specialty alcohol treatment in the overall sample (AOR: 1.49, 95% CI: 1.14–1.94) compared to having health insurance of any kind. However, it was not significantly associated with experiencing a gap in specialty drug treatment services for Black men or Black women specifically.

## Discussion

To our knowledge, this is the first study to examine predictors of specialty drug and alcohol treatment gaps specifically among Black Americans. We found evidence in our overall sample that Black bisexual adults experience significantly greater gaps in specialty drug and alcohol treatment services compared to their heterosexual peers. However, gender-stratified analyses showed significantly greater odds of specialty alcohol and drug treatment utilization for Black bisexual women but not bisexual men. Furthermore, we found that lesbian Black women experience similar gaps compared to heterosexual Black women.

Despite finding only partial support of our first hypothesis, our results are consistent with the broader literature demonstrating that sexual minority individuals experience greater disparities in accessing and utilizing specialty substance use treatment services. Scholars have such as Fisher and colleagues [46] found that sexual minority adults experience more challenges with gaining access to substance use treatment services relative to heterosexuals, particularly those individuals who attempted to gain access for the first time. From an intersectionality perspective, the partial support for the hypothesis may be explained by counterintuitive findings that are often reported in the scientific literature [35]. Our inclusion of social determinants of health in our analysis (e.g., poverty status, education, social class) is consistent with scholars who have argued intersectionality theory as applied to population research must extend beyond identity by including social positions, policies, and other ecological structures [47]. Overall, our results suggest that drug and alcohol treatment gaps within Black populations may be more nuanced and complex than previously reported. Furthermore, sexual minority stressors and the intersection of multiple historically marginalized identities may have a strong effect on substance use treatment gaps, independent of other social determinants of health.

We also found support for our second hypothesis in that having no health insurance was a significant predictor for experiencing a drug or alcohol specialty treatment gap for Black men and women. Although we did not examine type of health insurance, scholars have found that having health insurance such as Medicaid is associated with receiving substance use treatment services [48]. However, Black Americans continue to experience lower rates of Medicaid coverage even after the Affordable Care Act became law [28, 49]. As such, it remains critical to implement statewide and federal policy changes to ensure equitable access to health insurance that support substance use treatment services for Black Americans.

Black men with no health insurance were significantly more likely to experience a specialty drug treatment gap compared to those with health insurance. This disparity may be due to the syndemic effects of existing structural racism and classism that perpetuate disparities in substance use utilization [50–52]. Prior research has also shown that Black sexual minority men experience even greater disparities in having health insurance than their heterosexual peers [53]. For Black men with a lack of health insurance, implementing programs and policies that ensure their equitable access to substance use treatment services may mitigate some of the socioeconomic barriers that perpetuate such disparities [54].

### Limitations and Conclusions

Several limitations warrant cautious interpretation of our results. The cross-sectional nature of the study prevents us from establishing causal relationships. Additionally, the self-report nature of the NSDUH increases the risk for recall bias, such that individually reported rates of misuse may be an underestimation. Furthermore, this analysis did not account for factors such as spirituality and cultural identity that may influence Black Americans’ decisions to utilize in substance use treatment services. The NSDUH does not provide data on gender identity (e.g., transgender, genderqueer, nonbinary), which limits the extent to which other intersectional minority stressors may influence treatment gaps. Moreover, the wide 95% confidence intervals reported for the bisexual subsample warrants interpretation of the adjusted odds ratios with extreme caution. The substantial percentage of missing data for sexual orientation highlights a greater need for investigations that are more intentional in recruiting Black sexual minority adults using culturally responsive methods.

Despite the limitations, the results highlight differences within and across Black populations that have not, to our knowledge, been fully examined. As such, this study offers avenues for future practice implementation. First, more tailored outreach and interventions rooted in intersectionality and minority stress may mitigate some of the barriers to care. Developing participatory interventions and educational outreach campaigns that speak to the experiences of Black LGBTQ + individuals can help ensure that substance use treatment efforts address the systems of oppression that contribute to disparities in misuse. Second, creating or modifying specialty substance use treatment programs that integrate an Africentric approach [55] may provide Black Americans with more culturally responsive options for care. For example, such programs can integrate concepts of Black womanhood, affirming age, gender identity, and sexual orientation into existing practices. Third, local, state, and federal policymakers can use the results to implement creative measures for uninsured Black Americans to receive necessary specialty substance use treatment services. Such measures may not only provide stronger mechanisms for implementing culturally responsive approaches to substance use treatment for Black Americans, but they may also aid in dismantling the systems of oppression that perpetuate minority stressors within existing models of care.

## Data Availability

All data and materials can be accessed through SAMHSA’s website at https://www.samhsa.gov/data/data-we-collect/nsduh-national-survey-drug-use-and-health.
